# An age-dependent branching process model for the analysis of CFSE-labeling experiments

**DOI:** 10.1186/1745-6150-5-41

**Published:** 2010-06-22

**Authors:** Ollivier Hyrien, Rui Chen, Martin S Zand

**Affiliations:** 1 Department of Biostatistics & Computational Biology, University of Rochester Medical Center, 601 Elmwood Avenue, Rochester, NY 14642, USA; 2 Department of Medicine, Division of Nephrology, University of Rochester Medical Center, 601 Elmwood Avenue, Rochester, NY 14642, USA

## Abstract

**Background:**

Over the past decade, flow cytometric CFSE-labeling experiments have gained considerable popularity among experimentalists, especially immunologists and hematologists, for studying the processes of cell proliferation and cell death. Several mathematical models have been presented in the literature to describe cell kinetics during these experiments.

**Results:**

We propose a multi-type age-dependent branching process to model the temporal development of populations of cells subject to division and death during CFSE-labeling experiments. We discuss practical implementation of the proposed model; we investigate a competing risk version of the process; and we identify the classes of cellular dependencies that may influence the expectation of the process and those that do not. An application is presented where we study the proliferation of human CD8+ T lymphocytes using our model and a competing risk branching process.

**Conclusions:**

The proposed model offers a widely applicable approach to the analysis of CFSE-labeling experiments. The model fitted very well our experimental data. It provided reasonable estimates of cell kinetics parameters as well as meaningful insights into the processes of cell division and cell death. In contrast, the competing risk branching process could not describe the kinetics of CD8+ T cells. This suggested that the decision of cell division or cell death may be made early in the cell cycle if not in preceding generations. Also, we show that analyses based on the proposed model are robust with respect to cross-sectional dependencies and to dependencies between fates of linearly filiated cells.

**Reviewers:**

This article was reviewed by Marek Kimmel, Wai-Yuan Tan and Peter Olofsson.

## Background

Carboxyfluorescein succinimidyl ester (CFSE)-labeling experiments have become a standard assay in the analysis of cell proliferation kinetics since Lyons and Parish [1] developed the technique. The assay has become widely used to investigate the processes of division and death of activated lymphocytes [2-13]. The popularity of this flow cytometry-based assay rests on the ability of the dye CFSE to track how many times any individual cell has divided since the beginning of the experiment. Using additional fluorescent markers attached to the cell membrane, to intra-cellular proteins, or to the nucleus, other cellular properties may be identified. For instance live and dead cells can be distinguished from each other using TOPRO-3, a fluorescent dye that binds to the DNA and RNA inside the plasma membrane of dead cells. The information obtained by combining multiple markers offers a means with unprecedented power to further advance our understanding of the most basic cellular functions (proliferation, death, and differentiation) and how these functions may be optimized or modulated by an external signal such as a treatment. The events of interest (division, differentiation, or death of individual cells) cannot be observed during CFSE-labeling experiments, and mathematical modeling offers an attractive approach to the quantitative analysis of cell kinetics in this setting. A number of mathematical models have been proposed to describe the proliferation and death of cells in successive generations, as observed during CFSE-labeling experiments. The utility of stochastic models has been investigated in several publications. For instance, Hawkins et al [9] proposed a model which they referred to as a *cyton*; Yates et al [11] considered a discrete time branching process; Hyrien and Zand [12] considered the utility of age-dependent branching processes; Lee and Perelson [13] investigated a Smith-Martin model. These models present various limitations. For example, the cyton model is formulated through a competing risk approach under which the probability of division is completely specified by the distributions of the time to division and of the time to death. The process proposed by Hyrien and Zand [12] did not include cell death because additional issues had to be addressed. One goal of the present paper is to propose a general stochastic model - built on the branching process proposed by Hyrien et al [14] - that mitigates these limitations. The proposed model contains many of the existing models as particular cases. For an overview on branching processes, we refer to [15-17].

### A motivating example: proliferation of CD8+ T lymphocytes

CD8+ T lymphocytes are responsible for killing cells infected with viruses. Such killing requires recognition of a foreign marker, typically a viral protein fragment presented on the cell surface by the Major Histocompatibility Complex (MHC) Class I proteins, which is then recognized by the cognate T cell receptor expressed on the surface of the CD8 T cell. CD8 T cells normally persist in a resting, non-dividing state in the lymph nodes, spleen, peripheral blood and tissues, until they become activated. Activation occurs with the binding of the cognate T cell receptor to the MHC Class I protein containing the foreign peptide fragment, along with several other accessory signals. Once activated, CD8 T cells multiply rapidly, usually within a lymph node, spleen, or mucosal associated lymphoid tissue. After a moderate but limited number of divisions, the activated CD8 T cells cease dividing. Some of these activated cells stay in the lymph node to become resting memory CD8 T cells, which can be activated again upon re-exposure to the viral antigen, for instance with re-infection. The majority of CD8 T cells, however, stop dividing, exit the lymph node, travel to the site of infection or inflammation in order to kill infected cells, and most cells ultimately die within several days. The effectiveness of CD8+ T cells in controlling the spread of an infection depends on their proliferation rate in relation to the rate at which the virus can reproduce. In particular, critical parameters for the success or failure of an immune response in controlling infection for a particular viral pathogen include: the time to activation, the time to division and the probability of division of activated CD8+ T cells. Multiple biological factors may modulate these parameters, including the type and strength of activating signals, or the local presence of cytokines which improve the efficiency of the division of CD8+ T lymphocytes, to name a few. The methods proposed in this paper may be used to quantify the effects of stimuli on these parameters using CFSE-labeling experiments.

### The principle of CFSE-labeling experiments

CFSE is a fluorescent dye that was first used by Lyons and Parish [1] as a means to track the division history of individual cells. In a typical CFSE-labeling experiment, a pool of cells is isolated, either by extraction from blood, a tissue or from a cell line, and then pulse-labeled through brief incubation in a CFSE-containing solution. A fraction of the dye binds non-specifically to intra-cellular proteins, and the remaining unbound dye solution is washed out. The cells are aliquoted into small wells in standard 96-well tissue culture plates at concentrations ranging typically between 10^4 ^- 10^6 ^cells per 200 *μ*liter well. In addition, in experiments involving lymphocytes, a stimulus is added to the culture medium in each well so cells may engage in division. The cells are then sampled at various times after the start of the experiment.

The amount of CFSE contained in individual cells can then be quantified by flow cytometry, a technique by which fluorescently tagged single cells are suspended in a fluid stream, passed through an optical cell where a laser excites the CFSE dye, and a photomultiplier measures the total fluorescence emission from the dye in the (biological) cell. This technique allows measurement of fluorescence in up to 10^7 ^individual cells in a single sample, with multiple samples run for a single experiment.

**Figure 1:**
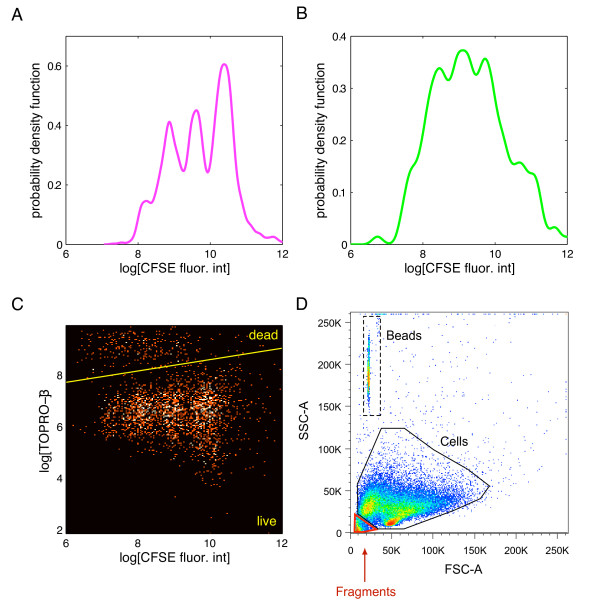
**Data collected during CFSE-labeling experiments**. Kernel density estimates of the log-transformed CFSE fluorescence intensity in live (Panel A) and in dead (Panel B) CD8+ T lymphocytes 64 hours after the start of the experiment. Each identifiable peak corresponds to one generation. The peaks are less distinguishable in dead cells than in live cells. Panel C shows a scatterplot of the log-transformed TOPRO-3 fluorescence intensity vs. the log-transformed CFSE fluorescence intensity in thousands of individual CD8+ T cells; the pool of cells expressing high TOPRO-3 are the dead ones, and the remaining cells are considered as alive. Panel D: scatterplots of side and forward scatters; examination of the plot enables to distinguish between cells, debris, and beads as shown.

When a cell divides, the CFSE molecules that it contains are partitioned in approximately equal amount between each daughter cell, causing the CFSE-fluorescence intensity to decrease by a factor of two in each generation. When the histogram (or any other estimate of the probability density function (p.d.f.)) of the log-transformed CFSE-fluorescence intensity is plotted, the identifiable peaks are indicative of the generation numbers, with the peak with highest fluorescence intensity corresponding to cells of first generation (that is, undivided cells), the second highest peak corresponding to cells of second generation (cells having divided once), etc. The p.d.f. of the CFSE-fluorescence intensity evolves over time in accordance with the kinetics of the cells. Examples of CFSE-labeling data are displayed in panels A-B of Figure 1. The data are dependent because the fluorescence intensities of cells that arise from the same ancestor cell are all related to the amount of CFSE contained in the common parent cell (see [12]).

Under typical experimental conditions, cells also undergo apoptosis or programmed cell death. When a cell dies, its plasma membrane becomes permeable to certain classes of fluorescent dyes. TOPRO-3 is one such dye that binds to the RNA and DNA, which normally remain shielded in living cells by the intact plasma membrane. Similarly to CFSE, TOPRO-3 emits light upon excitation by a laser. The intensity of the resulting fluorescence can be measured by flow cytometry, and used to distinguish between live and dead cells. Thus, by using simultaneous CFSE and TOPRO-3 labeling on the same cells, one can separate live and dead cells in multiple generations of divided cells, as shown in panel C of Figure 1. This is accomplished by using a threshold: cells are declared dead if they fall below this threshold, and live otherwise. This approach is reasonable here since the fluorescence intensities of live and dead cells appear to have little overlap.

Dead cells *in vitro *eventually disintegrate or fragment. The scattering properties of the fragments allow distinguishing them from intact, non-fragmented cells. The important implication of disintegration is that the absolute number of cells that have died by any given time is not experimentally measurable, and models should include this feature.

For sampling and analysis, it is often the case that multiple wells are pooled together so each experimental group contains cells in sufficient number for the measurements to be taken. The cells are then washed, incubated with TOPRO-3, and washed again. An approximately known number of fluorescent beads is also added to the cell suspension. These beads are used to back-calculate the total number of cells in an entire cell group. Each experimental group thus contains a pool of cells and beads run on the low cytometer. A number of "*events*" are collected for each experimental group, generally 10^5 ^- 10^6 ^events, with an event being defined as a set of flow cytometric measurements collected on a single object (that is, a cell, a bead or debris). Each set of measurements includes two parameters of light scattering properties (forward scatter - FSC, and side scatter - SSC) and the fluorescence intensity of CFSE and TOPRO-3 (and possibly of additional protein markers labeled by fluorescent dyes as well). Bivariate plots of the light scattering properties of the objects can then be used to distinguish between beads, cells and debris, and to count the number of each (panel D of Figure 1).

Let  denote the total number of cells contained in any given group of wells at time of sampling, and let denote the number of beads added to these cells. Only a fraction of the pool of cells and beads in any experimental group is sampled by the flow cytometer. Various sampling strategies exist. One of them consists in letting the flow cytometer sample objects until a pre-specified number of beads, say , has been recaptured. This sampling scheme is known as a capture-recapture experiment with single-mark release [18]. Let  denote the number of cells sampled along with the recaptured beads. The total number of cells in the experimental group can be estimated as , which is the Petersen estimator [18].

## Results

### A multi-type age-dependent branching process

This section presents a model of the temporal development of a cell population through successive generations (as observed during CFSE-labeling experiments). Our model accounts also for the processes of division, death and disintegration possibly encountered by every cell. It is built using an extension of the multi-type Bellman-Harris branching process formulated by Hyrien et al [14] (see also [19-21]) and which allows the distributions of the time to cell transformation (e.g., division or death) to depend on the transformation ultimately undergone by the cell. It is formulated as a multi-type age-dependent branching process where the type of each cell is defined by two characteristics: firstly, a *generation*, defined as the number of times the cell has divided since the start of the experiment +1; and secondly, a *status *that corresponds to whether the cell is alive or dead. Any cell of given generation *g *and status *s *shall be referred to as a type-(*g*, *s*) cell. Every cell ultimately transforms (that is, divides, dies, or disintegrates, depending on the type of the cell), and we shall refer to the time that is necessary for any cell to complete its transformation as its *lifespan*, irrespective of the nature of the transformation (division, death or disintegration). Likewise, *offspring *shall refer to the pool of new cells resulting from the transformation of the cell, whether these new cells are alive or dead.

**Figure 2:**
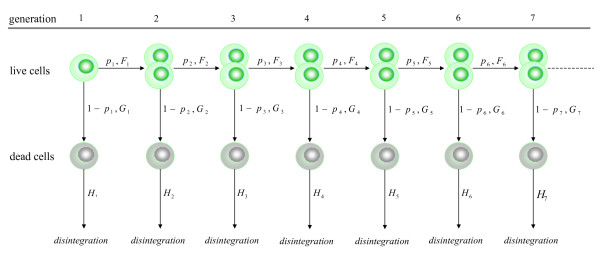
**Structure of the general model**. Diagram showing the structure of the general branching process model.

The structure of the proposed branching process is schematized in Figure 2. It begins with a random number *N*_0 _of initiator cells, all alive and of generation 1 (that is, type-(1,1) cells). Upon completion of its lifespan, every live cell (*s *= 1) of generation *g *(*g *= 1,2⋯) will either divide into two new live cells of generation *g *+ 1 with probability *p*_*g *_(equivalently said, it will produce two type-(*g *+ 1, 1) cells), or it will die and turn into a single type-(*g*, 0) cell with probability 1 - *p*_*g*_. Upon completion of its lifespan, every dead cell (*s *= 0) of generation *g *(*g *= 1,2⋯) will ultimately disintegrate and disappear from the population.

Let the random variable (r.v.) *ξ*_*g*,1 _denote the transformation undergone by any live cell of generation *g*, with *ξ*_*g*,1 _= 1 with probability *p*_*g *_= *P*(*ξ*_*g*,1 _= 1) if the cell divides, and *ξ*_*g*,1 _= 0 with probability 1 - *p*_*g *_if the cell dies. Past experimental studies [14,19,21] and the analysis of our experimental data (see below) suggested that the distribution of the cell lifespans could differ substantially depending upon the type of transformation undergone by the cells. In some instances, the time required for the cells to reach division, calculated from their birth, could be (stochastically) shorter or longer than their time to death or than their time to differentiation when such a possibility exists. Taking this finding as a generally applicable principle, we define two cumulative distribution functions (c.d.f.) for each generation of live cells: the first one, denoted as , is the c.d.f. of the time to division ; the second one, denoted as , is the c.d.f. of the time to death . These distributions are possibly dissimilar. The lifespan of any live cell of generation *g*, denoted as *τ*_*g*,1_, can be represented as

Notice also that

and the c.d.f. of *τ*_*g*,1 _is therefore the mixture *p*_*g*_*F*_*g*_(*t*) + (1 - *p*_*g*_)*G*_*g*_(*t*). In assuming this formulation for the distribution of *τ*_*g*,1_, we allow the lifespan and the offspring of any live cell to be dependent, thereby extending the Bellman-Harris branching process to which our model reduces if the conditional distributions are all identical; that is, if *F*_*g*_(*t*) = *G*_*g*_(*t*) for all *g *= 1,2⋯. Lastly, the time to disintegration of any dead cell of generation *g *is modeled as a r.v. *τ*_*g*,0 _with c.d.f. *H*_*g*_(*t*) = *P*(*τ*_*g*,0 _≤ *t*), and every cell evolves independently of every other cell. We shall see later on that this assumption can be somewhat relaxed.

Let *Z*_*g*,*s*_(*t*) denote the number of type-(*g*, *s*) cells in the population at any time *t*. Write  for the total number of cells, and ∏_*g,s*_(*t*)= *Z*_*g,s*_(*t*)/*Z*^#^(*t*) for the proportion of type-(*g*, *s*) cells, both at time *t*. Define the associated conditional expectations, *m*_*g,s*_(*t*) = *E*{*Z*_*g,s*_(*t*)|*Z*^#^(0) = 1} and *m*^#^(*t*) = *E*{*Z*^#^(*t*)|*Z*^#^(0) = 1}, and introduce the conditional expectation of the proportion of type-(*g*, *s*) cells at time *t*, given the population has not died out by time *t*: *π*_*g,s*_(*t*) = *E*{∏_*g,s*_(*t*)|*Z*^#^(*t*) > 0}. Define the convolution of the c.d.f. of the times to division of the first *g *generations , where  denotes the convolution of two c.d.f.s *F*_*g*1 _and *F*_*g*2_. By convention, we shall write .

Let **u **= (*u*_*g*,*s*_; *s *= 1,2; *g *= 1,2,⋯), and introduce the probability generating function

where **1**_{·} _denotes the indicator function. It is not difficult to show that the functions Ψ_*g,s*_(**u**, *t*), *s *= 1, 2 and *g *= 1,2⋯, satisfy the set of functional equations

and

respectively. By differentiating both sides of the above equations with respect to **u**, and using a little algebra, one can show that the expected number of live cells and of dead cells of generation *g *at time *t *are respectively given by(1)

and(2)

By the Law of Large Numbers and the independence assumption, we have (as *N*_0 _gets large) that *Z*_*g,s*_(*t*)/*N*_0 _= *m*_*g,s*_(*t*) + *o*_*p*_(1) and *Z*^#^(*t*)/*N*_0 _= *m*^#^(*t*) + *o*_*p*_(1), so that ∏_*g,s*_(*t*) = *m*_*g,s*_(*t*)/*m*^#^(*t*) + *o*_*p*_(1). We shall therefore approximate the conditional expectation *π*_*g*,*s*_(*t*) by the ratio of the expectations(3)

This approximation is appropriate and justified for practical purpose because the number of cells at time 0 in CFSE-labeling experiments is typically very large (*N*_0 _≫ 10^5 ^or 10^6^).

One of the primary endpoints measured during CFSE-labeling experiments is the CFSE-fluorescence intensity of individual cells. As mentioned earlier, the distribution of this endpoint changes over time in accordance with the kinetics of the cell population. We relate our branching process to the dynamic of this marker by proceeding as follows. Let *Y*(*t*) denote the log-transformed CFSE-fluorescence intensity measured in any given cell at time *t*, and let *f*(*y*; *t*) denote the marginal p.d.f. of *Y*(*t*). Using the Law of Total Probability this p.d.f. can be expressed as a mixture , where *f*_*g*,*s*_(*y*; *t*) denotes the p.d.f. of the CFSE-fluorescence intensity of type-(*g*, *s*) cells at time *t*. We shall therefore approximate *f*(*y*; *t*) by the mixture distribution(4)

with  given as in equation (3). Let *μ*_*g*,*s*_(*t*) = ∫_***R***_*yf*_*g*,*s*_(*y*; *t*)*dy *denote the mean log-CFSE fluorescence intensity of any cell of generation *g *and status *s*. Under the assumption that the CFSE-fluorescence intensity of any cell is halved at each division, we have that *μ*_*g,s*_(*t*) = *μ*_*g*+1,*s*_(*t*) + log 2.

### A competing risk branching process

A particular case of our model arises naturally from a competing risk approach. Let *λ*_*g*,0 _and *λ*_*g*,1 _denote two independent r.v.s with respective c.d.f.s *L*_*g*,0 _and *L*_*g*,1_. One may think of *λ*_*g*,0 _and *λ*_*g*,1 _as two latent, competing failure times. The smaller *λ*_*g*,*s *_determines both the lifespan of the cell and whether the cell divides or dies; that is, *τ*_*g*,1 _= min{*λ*_*g*,0_, *λ*_*g*,1_}, and *ξ*_*g*,1 _= 1 iff *λ*_*g*,1 _≤ *λ*_*g*,0_, or *ξ*_*g*,1 _= 0 iff *λ*_*g*,0 _≤ *λ*_*g*,1_. Clearly, the probability of division is given by(5)

Notice that the time to division  is equal to *λ*_*g*,1 _conditional on the event {*λ*_*g*,1 _≤ *λ*_*g*,0_}, and, similarly,  = *λ*_*g*,0 _conditional on {*λ*_*g*,0 _≤ *λ*_*g*,1_} for the time to death. The c.d.f.s of the time to division and of the time to death are therefore given by *F*_*g*_(*t*) = *P*(*λ*_*g*,1 _≤ *t*|*λ*_*g*,1 _≤ *λ*_*g*, 0_) and *G*_*g*_(*t*) = *P*(*λ*_*g*,0 _≤ *t*|*λ*_*g*,0 _≤ *λ*_*g*, 1_), and are related to *L*_*g*,0 _and *L*_*g*,1 _through the identities(6)

This competing risk process is an age-dependent linear birth-and-death process that was proposed by Waugh [22,23]. Hawkins et al's cyton model [9] was built in that spirit. This formulation is theoretically appealing, but the resulting process has some important limitations when applied to cell biology.

Specifically, the model assumes that the decision made by any cell to divide or to die and the actual event (division or death) occur simultaneously. This assumption would not be appropriate if any cell needs additional time to complete its transformation from the time its decision to divide or to die has become irreversible (e.g., the time to complete mitosis or undergo programmed cell death). Furthermore, the intricate relationship induced by the latent r.v.s between the probability of division, the distributions of the time to division, and that of the time to death may prevent the resulting process from capturing salient features of cell proliferation kinetics.

As an illustration, consider the case where the latent r.v.s *λ*_*g*,0 _and *λ*_*g*,1 _are exponentially distributed. Denote the parameters of these distributions by *θ*_*g*,0 _and *θ*_*g*,1_, respectively. It follows from equations (5) and (6) that *p*_*g *_= *θ*_*g*,1_/(*θ*_*g*,0 _+ *θ*_*g*,1_) and *F*_*g*_(*t*) = *G*_*g*_(*t*) = 1 - *e*^-(*θ*_*g*,0 _+ *θ*_*g*,1_)*t*^. Thus, in this example, the competing risk formulation leads to a Bellman-Harris process and it prevents the time to division and the time to death from having dissimilar distributions, which may be an undesired modeling feature in cell kinetics studies. The more general process that we consider in this paper would allow the time to division and the time to death to follow dissimilar exponential distributions, while permitting the probability of division to take freely any value within the interval [0, 1].

The limitation of the Markov competing risk process could be somehow mitigated by using more flexible distributions for *L*_*g*,0 _and *L*_*g*,1 _(such as gamma distributions). However, the resulting model would still implicitly assume a specific relationship between *p*_*g*_, *F*_*g*_, and *G*_*g*_. The nature of this relationship will remain, in general, difficult to determine explicitly because, for instance, the calculation of *F*_*g *_and *G*_*g *_involves truncated bivariate distributions. One of the simplest biologically relevant cases is when the latent r.v.s *λ*_*g*,0 _and *λ*_*g*,1 _are both log-normally distributed. Denoting their respective parameters by *μ*_*l *_and , *l *= 0, 1, (assuming they do not depend on *g*), a simple calculation shows that

where Φ denotes the c.d.f. of the standard normal distribution. Thus, the probability of division is constrained by the parameters of *L*_*g*,0 _and *L*_*g*,1_; in particular, it is greater or smaller than 1/2 depending on the sign of *μ*_0 _- *μ*_1_. Also, it follows from the theory of skew-normal distributions [24] that the density function of , denoted and defined as *f*_*g*_(*t*) = *dF*_*g*_(*t*)/*dt*, is explicitly given by(13)

where *Φ *denotes p.d.f. of the standard normal distribution, and where . The calculation of *F*_*g*_(*t*) requires numerical integration, however. The limitations of competing risk branching processes will also appear in our analysis of experimental data on CD8 T cells.

### Computing the moments of the process

The expectations *m*_*g*,*s*_(*t*) can be expressed as linear combinations of functions that take the form , where *k *= 1,2⋯ and where *k*_*G*_, *k*_*H *_= 0,1. These moments may be computed explicitly in some special cases, but it otherwise requires the use of approximation techniques. To simplify notation, we shall write *C*_**K**_(*t*) in place of , where **K **= (*k*, *k*_*G*_, *k*_*H*_). One approach is to use the technique of saddlepoint approximations, as proposed by Hyrien et al [19], which applies for a variety of distributions. Assume for instance that *F*_*k*_, *G*_*k*_, and *H*_*k *_are gamma distributions. Let *μ*_*F*,*k *_= *α*_*F*,*k*_*β*_*F*,*k *_and  (resp. *μ*_*G*,*k *_= *α*_*G*,*k*_*β*_*G*,*k *_and ; *μ*_*H*,*k *_= *α*_*H*,*k*_*β*_*H*,*k *_and ) denote the mean and variance of *F*_*k *_(resp. *G*_*k*_; *H*_*k*_). Write  and  for the mean and variance of the distribution function *C*_**K**_. The saddlepoint approximations to the expectations *m*_*g*,1_(*t*) and *m*_*g*,0_(*t*) are given by

where(7)

where

and where  is the root to the equation , which can be solved explicitly in some cases, or, when not possible, numerically. We refer to [19] for further details on the construction of the approximations in the case of gamma distributions and for other distributions as well.

An alternative approach consists of using Monte Carlo integration, where the unknown functions *C*_**K**_(*t*) are replaced by empirical estimators obtained by simulating r.v.s from the distributions *F*_*k*_, *G*_*k*_, and *H*_*k*_. The approach is straightforward and not described further.

### Effects of cellular dependencies

The presence of dependencies among cells of a same family tree has long been reported in the literature [21,25-27]. One of the main type of dependency identified in these studies was found between the lengths of the mitotic cycle of sister cells. This type of dependency is known to have no effect on the expectation of branching processes. This property was established by Crump and Mode [28] for a binary splitting process, and can be extended to the branching process under consideration herein (see below). More general dependency structures may take part in the evolution of populations, as discussed by Olofsson [29], including the possibility that fates of siblings be dependent (for instance, sister cells would both tend to divide or die). Some authors have considered cellular dependencies that arise from the regulation of cell size [30]. A general class of cellular dependencies that has received much interest in past studies arises when all cells of a clone inherit specific properties from the founding cell. Stivers, Kimmel, and Axelrod [31] investigated branching process models of the inheritance of cell lifespans, whereas Boucher et al [32] and Hyrien et al [19] proposed branching process models for the inheritance of cell fate. In the latter case, the models assumed that all cells of a same clone must undergo a pre-determined number of divisions (referred to as "critical number") before they become competent for differentiation (or death). Cells of prior generations would all divide. The intra-clone dependencies in cell fate is induced by letting the number of critical cycles vary randomly across clones. This idea has been used recently by Wellard et al [33].

In what follows we identify the dependencies that may influence the expectation of the number of cells, and obtain a general expression for the expectation of the process that remains valid in the presence of dependencies. To do this, notice first that any generation may consist of up to 2^*g*-1 ^cells (either live or dead). For every *g *= 1,2⋯, *s *= 0, 1, and *a *= 1,...,2^*g*-1^, let *χ*_*g*,*s*,*a*_(*t*) denote an indicator r.v. equal to one if the *a*th type-(*g*, *s*) cell exists in the population at time *t*. The process *Z*_*g*,*s*_(*t*) can be decomposed as

Let  be a r.v. denoting the time of birth of any type-(*g*, *s*) cell, and let  denote the time at which this cell produces its progeny (that is, either divides, dies, or disintegrates). Notice that  and  can be represented as(8)

Let *φ*_*g*,0 _and *φ*_*g*,1 _denote events defined as

When conditioning on *φ*_*g*,1_, it is easy to see that the decomposition of  given in equation (8) specializes to , whereas that of  conditional on *φ*_*g*,0_, becomes , where  denotes a collection of (here) possibly dependent r.v.s all drawn from the distributions of the times to division, and where . Write

and

Since

we have

To develop further the expression for *q*_*g*,1_(*t*), notice that(24)

Denoting the complement of *φ*_*g*,1 _by , let(25)

Conditional on , the r.v.s *ξ*_*g*,1 _and *χ*_*g*,*s*,*a*_(*t*) are mutually independent, so that the conditional distribution of *ξ*_*g*,1_, given , can be chosen arbitrarily without altering the expectation of *χ*_*g*,*s*,*a*_(*t*). In particular we can set . Under this particular choice, *ξ*_*g*,1 _and *φ*_*g*,1 _are mutually independent, and we have that

and

Therefore, we deduce that

Using similar arguments, we can show that

where

We deduce from the above derivations the following alternative expressions for the expectation of the processes *Z*_*g*,*s*_(*t*) and *Z*^#^(*t*), and a property in the presence of dependencies.

**Proposition 1. ***The expectations of Z*_*g*,*s*_(*t*) *and Z*^#^(*t*) *admit the general form*

*Furthermore, these expressions remain valid when the independence assumptions regarding the evolution of age-dependent branching processes are relaxed*. ◇

These expressions resemble closely those obtained under the independence assumption (see equations (1) and (2)). The only noticeable difference is that the convolutions (e.g., ) are replaced by the c.d.f.s of sums of possibly dependent r.v.s (e.g., ).

It follows from Proposition 1 that the mean number of cells is not affected by *cross-sectional *dependencies (that is, by dependencies existing between cells that are not *linearly filiated*, such as cousins and aunts). In other words, in the presence of this type of dependencies alone, the expressions for *m*_*g*,1_(*t*) and *m*_*g*,0_(*t*) will simplify to those of equations (1) and (2). For instance, any form of dependency existing between the lifespans of sister cells (as previously shown by Crump and Mode [28]) will have no effect on the expectation of the process. Likewise dependencies between the fates of sister cells will not change the expectation of the process either. The property extends to the case where the fate/lifespan of any cell are dependent with those of its cousins (of any degree), with those of its aunts (or the cousin of its aunts), etc. Furthermore, the presence of dependencies between the fates of linearly filiated cells has no effect on the expression for the expectation. Thus, any mother-daughter correlation in fate will leave the expectation of the process identical to that obtained under the independence assumption. The only possible changes in the expectation of the process are those induced by dependencies between the lifespans of linearly filiated cells. Using the above line of arguments, it is also easy to see that higher order moments (e.g, the variance) of the process will generally be affected by the presence of cross-sectional dependencies and by dependencies between linearly filiated cells. This is a well-known result also due to [28]. Since the variance of the process does not play any role in the analysis of CFSE-labeling experiments, the effects of cell dependencies on this characteristic of the process are not relevant in the context of CFSE-labeling data.

### An application to human CD8+ T lymphocytes

**Figure 3:**
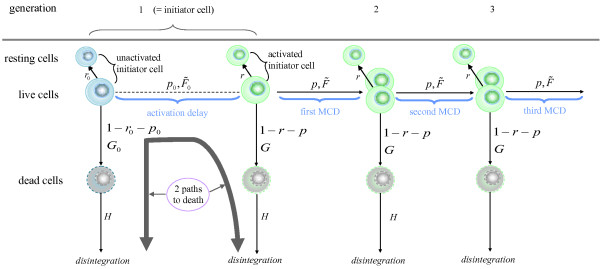
**Structure of the model for the proliferation of CD8 T cells**. Diagram showing the structure of the branching process that describes the proliferation and death of CD8+ T lymphocytes; MCD = mitotic cycle duration; , , ,  and  denotes the c.d.f of the various times to event;  and  denote probabilities of death;  denotes the probability of activation of unactivated initiator cells;  denote probabilities of division of activated cells; and  and  denote probabilities of resting.

We analyzed a set of experimental data for the proliferation of human CD8+ T lymphocytes. The structure of the model used to describe the proliferation and death of lymphocytes is illustrated in Figure 3. When the experiment begins, the initiator lymphocytes are unactivated (or resting) and they do not divide. A fraction of these initiator cells will eventually become activated upon stimulation (which we accomplished in our experiment using anti-CD3 plus anti-CD28 antibodies). The remaining non-activated cells will either stay resting or die. Once activated a cell may either divide, or die, or return to a resting state permanently; this occurs in every generation. Thus, there exists two possible paths to death for initiator cells, as indicated in Figure 3. In the model formulated below we distinguish activated and unactivated cells. Once it is defined, we shall explain the correspondence between this model and the branching process formulated earlier. In line with the above description, we shall assume that

(A1) Upon completion of its lifespan, every unactivated initiator cell either dies with probability , or becomes activated with probability , or remains unactivated (or resting) indefinitely with probability . By convention, activated and unactivated initiator cells are said to be of generation 1. The time to death of any unactivated initiator cell is described by a non-negative r.v. with c.d.f. , whereas the time to activation is modeled as a non-negative r.v. with c.d.f. .

(A2) Upon completion of its lifespan, every live, activated initiator cell of generation *g *= 1,2⋯ either dies with probability , or divides into 2 new cells of age zero and generation *g *+ 1 with probability , or returns to rest (indefinitely) with probability . The time to death of any live, activated initiator cell of generation *g *is described by a non-negative r.v. with c.d.f. , whereas their time to division is modeled as non-negative r.v. with c.d.f. . The experimental setup does not distinguish activated/non-resting from unactivated/resting cells, making the distribution of the time that is necessary for a cell to return to its resting state non-identifiable. Therefore, we do not model it, and we assume that cells go back to rest instantaneously upon birth. The probabilities ,  and , and the c.d.f.s  and  are independent of the generation.

(A3) Resting cells have an infinite lifespan.

(A4) The time to disintegration of death cells of any generation *g *= 1,2⋯ is modeled as a non-negative r.v. with c.d.f.  that does not depend on the generation.

**Remark. **Even though the model assumes that the cells evolve independently of each other, we know from Proposition 1 that any dependencies between cell fates and any cross-sectional dependencies between lifespans will not alter the expression for the expectation of the process. Therefore the only assumption that we are making on the dependency structure is that the lifespans of linearly filiated cells are independent.

The above-formulated branching process is a particular case of the general model defined earlier. Treating resting cells as cells that would divide or that would become activated after an infinitely long lifespan, the c.d.f. of the time to division of cells of any generation *g*, with *g *= 2,3⋯ (in the general model) is given as an improper distribution

Likewise, one can show that *G*_1 _is the c.d.f. of a two-component mixture distribution

in which each component corresponds to one of the two possible paths that lead initiator cells to death, whereas the c.d.f. of the time to division, *F*_1_, is the convolution of  and , yielding

which is also improper. We also have *G*_*g *_= , *g *= 2,3,⋯, and *H*_*g *_= , *g *= 1,2⋯. Finally the probabilities of division (of the general model) are given by  and *p*_*g *_= 1 - , *g *= 2,3⋯. The expected cell counts and proportions of cells per generation under the model of the proliferation and death of CD8+ T cells are obtained using equations (1-3).

The c.d.f.s , , , and  were taken from the family of gamma distributions, and  was taken as an exponential distribution. The log-transformed CFSE-fluorescence intensity was modeled as a mixture of normally distributed r.v.s (the resultant fit suggested that this assumption was reasonable). The model was fitted simultaneously to the CFSE-labeling data and to the estimated cell counts using a modification of the method proposed by Hyrien and Zand [12] and using a pseudo-likelihood function proposed by Hyrien [34] and Hyrien et al [14,35].

**Figure 4:**
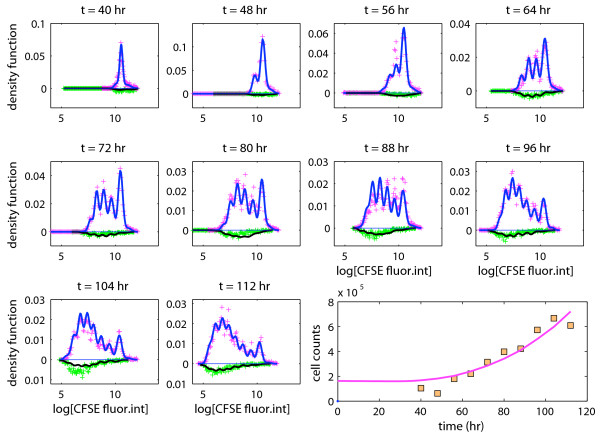
**The full model**. Experimental data and a fitted branching process: proportions of observations per bin (+) in live cells (positive values) and in dead cells (negative values); the lower right panel shows the estimated cell counts as a function of time.

**Figure 5:**
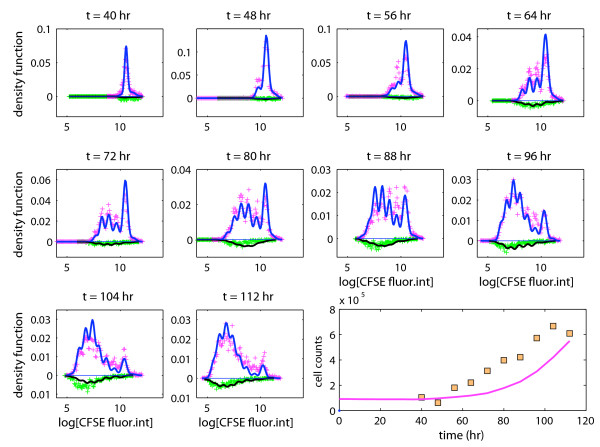
**A model where activated cells do not return to a resting phase**. Experimental data and a fitted branching process model that does not allow activated cells to return to a resting state. The fit of this simpler model was substantially poorer than that of the full model.

Figure 4 shows the histograms for the log-CFSE fluorescence intensity in live cells and in dead cells separately for all time points alongside the p.d.f. of the fitted mixture model displayed as solid lines. Also shown are the total cell counts in the wells estimated using the bead-based capture-recapture experiment as a function of time, and the corresponding fitted expected cell counts calculated from the branching process. The model provided a very good fit to the experimental data (except at 88 hours). The results of our analysis can be summarized as follows:

(C1) *Probability of death, activation, division, and resting. *Any unactivated, initiator cell would either die with probability (estimated as) , or become activated with probability , or remain indefinitely unactivated/resting with probability . Activated (non-resting) cells of generation 1 or greater would either die with probability , or divide with probability , or revert to a resting state with a probability , suggesting that a non-negligible fraction of the cells in each generation exited the mitotic cycle. The conditional probability of division, given that the cell does not go back to rest, was estimated as , which is in accordance with the size of the cell population increasing over time.

(C2) *Time to activation, division, death, and disintegration. *The mean and standard deviation of the time to activation were estimated as 47. 2 and 22. 6 hours, respectively. The mean time to division of activated cells was estimated as 12.9 hours. The estimated standard deviation of the time to division was relatively small (1.6 hours). The time to death of initiator cells that do not get activated was estimated as 0.15 hours. This suggests that a fraction of the initiator cells was already dead when placed in the wells, which is a reasonable finding and such death often occurs during the cell isolation step. The time to death of activated cells was estimated as 1.5 hours (estimated standard deviation ≃ 2.2 hours). Thus, the time to death was much shorter than the time to division, and indicated that dying cells were susceptible to labeling with TOPRO-3 shortly after birth. Dead cells would disintegrate after approximately 45 hours on average.

(C3) *Probability of resting. *The proposed branching process offered a good fit to the data, and we investigated whether a similar fit could be obtained using the simpler model that does not allow cells from returning to a resting state once activated; that is, we set  = 0 in the full model. The corresponding model fit (Figure 5) clearly suggests that inclusion of this parameter improves substantially the description of the data.

### Data analysis using a competing risk branching process

**Figure 6:**
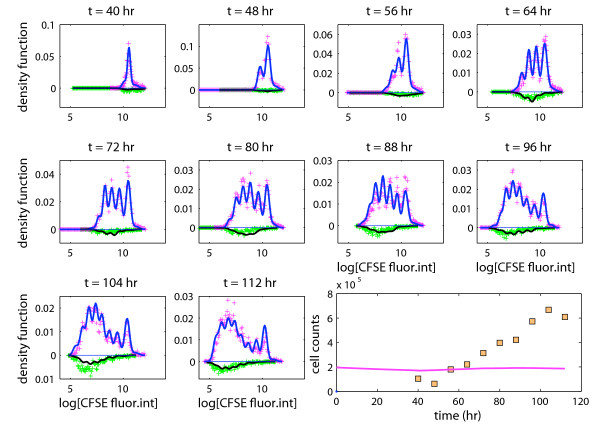
**A competing risk branching process**. Experimental data and a fitted competing risk branching process model. The model fails to capture the expansion of the population size over time (see bottom right panel).

Another simplified version of the model is defined by adopting a competing risk approach. In this alternative model the conditional probability of division of any activated cell, given the cell does not go back to rest,  say, was specified as , with *λ*_*g*,0 _and *λ*_*g*,1 _being the associated latent r.v.s assumed gamma distributed. The fitted competing risk branching process is shown in Figure 6.

Although this alternative model fitted the CFSE data almost as well as our full (unconstrained) model, it was clearly in poor agreement with the estimated cell counts, and it was unable to capture the continued expansion of the cell population over time (see bottom right panel in Figure 6).

When fitting our full model, we reached two conclusions: firstly, the times to death of activated CD8 T cells were much shorter than their times to division (1.5 vs. 12.9 hours; see (C2) in previous section); secondly, the probability of division of non-resting cells was estimated as 0.83. As explained below these two conclusions are difficult to reach simultaneously under the competing risk branching process. On the one hand, in order for the time to death to be (stochastically) substantially shorter than the time to division under the competing risk model, the latent r.v. *λ*_*g*,0 _should be shorter than the latent r.v. *λ*_*g*,1 _with a probability close to 1. Therefore, it follows from equation (5) that the probability of division would be close to 0 in such circumstances, thereby preventing the competing risk branching process from capturing the continued expansion of the cell population over time, as observed in our experiment. On the other hand, the competing risk branching process could capture an increase of the population size by letting the latent r.v.s *λ*_*g*,0 _and *λ*_*g*,1 _be such that . Informally speaking, this would imply that *λ*_*g*,1 _is, in some sense, stochastically shorter than *λ*_*g*,0_, which would make it difficult for the time to death to be much shorter than the time to division, as suggested by the analysis based on our full model.

When fitting the competing risk branching process to our experimental data, we reached a compromise between the above two scenarios. Specifically, the mean and standard deviation of the time to division were respectively estimated as 14.3 and 19.8 hours, whereas those of the time to death were estimated as 14.7 and 21.3 hours, so that neither the time to death nor the time to division would be stochastically much shorter/longer than the other. Moreover, while the estimates of the mean mitotic cycle duration did not differ much under either model (14.3 vs. 12.9 hours), the competing risk model suggested that the mean time to death was much longer than that obtained using the full model (14.7 vs. 1.5 hours). The standard deviations were also much larger using the competing risk model. The conditional probability of division, given that the cell does not revert to a resting state, resulting from the competing risk formulation was estimated as . The difference between the estimated probabilities  and  is dramatic, and leads to quite different conclusions in terms of population dynamics. Finally, the mean disintegration time was estimated as 5.4 hours using the competing risk model, which is much shorter than the 45 hours that we obtained with the proposed branching process, and explains why the proportion of dead cells in each generation is still well represented by the competing risk process.

## Discussion

We proposed a general framework for the quantitative analysis of CFSE-labeling data resting on multi-type age-dependent branching processes. The proposed method can be extended in a number of ways. For instance, for the sake of simplicity, we did not consider cell differentiation in this paper. This could be accomplished by including additional cell types in the model. See [14,19,21] for some examples of branching processes that include this feature. The branching processes proposed in these publications allow the distribution of the time to differentiation to differ from those of the time to division and of the time to death and they do not resort to a competing risk approach for specifying the fate of the cells.

### Competing risk branching processes in cell kinetics studies

We found that the competing risk version of our model did not offer a satisfactory description of the kinetics of CD8+ T cells. It is therefore likely that the central assumption behind the competing risk model does not reflect properly the actual biology. This assumption states that two independent biological processes compete continuously to determine the ultimate fate of the cell (division or death), and that the decision and the realization of the fate happen simultaneously. Several biological studies indicate that it is unlikely that these two events happen at the same time. Terrano et al [36] have found that the molecular pathways linking cell death and mitosis are dependent by coupling via cycling-dependent kinase 1-mediated Bcl-xL/Bcl-2 phosphorylation, and activation of one pathway suppresses the other. In addition, the decision for a cell to undergo apoptosis may be determined in prior generations, contradicting the main assumption of the model. For example, Hawkins et al [37] used time lapse microscopy to individually observe CFSE-labeled activated B cell founders, and their progeny, recording division and death times. They found that cells which died underwent successive changes in cell volume observable in prior generations which were predictive of death in daughter cells of the next generation. These independent studies therefore corroborate the conclusion that we have reached regarding competing risk branching processes.

### Effects of cellular dependencies

We have shown that the presence of cellular dependencies will generally leave unaffected the expectation of the proposed branching process. Only dependencies existing between the lifespans of linearly filiated cells (such as mother-daughter correlation) may change the expression for the expectations. Dependencies between the fates of linearly filiated cells and/or cross-sectional dependencies (either between fates or lifespans), however, will not alter their expressions.

Cellular dependencies have been identified using time-lapse microscopy experiments where cell trees started from a single founder cell are followed up over time so the complete genealogy of the tree can be reconstructed [21,25-27]. Recent work by Hawkins et al [37] and Wellard et al [33] suggests that such dependencies may play a role in lymphocyte activation, division and death decisions. However, it is unclear whether dependencies that have been found in these clonal studies and those of cells in mass culture are directly comparable. In order to adequately track cells in time-lapse photography, they must be cultured sparsely, with little cell-cell interaction. Under *in vivo *conditions, lymphocytes are activated within lymphoid structures, where they are surrounded, at minimum, by a large number of similarly activated cells. *In vitro *stimulation conditions that do not allow close cell-cell contact can lead to very different cell behavior, and erroneous conclusions, as Huggins et al [38] have shown for B lymphocyte activation and proliferation. In fact, it is likely that cell-cell interactions (which perhaps can be viewed as cross-sectional dependencies) may attenuate such filial dependencies and lessen their effects on cell kinetics at the population level. Furthermore, it remains technically difficult to conduct large scale time-lapse studies *in vivo*, which cannot currently be performed in human subjects. For these reasons, although informative for identifying the potential existence of dependencies, time-lapse cinematography experiments will not necessarily offer additional useful information to CFSE-labeling experiments about cell kinetics in large population of cells, especially *in vivo.*

### Further quantitative insights into the kinetics of CD8 T cells

One key function of CD8 T cells is to kill infected cells during the anti-viral immune response. This immune response is characterized by a large proliferative burst, followed by the eventual death of most effectors at the end of the response, and the survival of a cohort of memory CD8 T cells. These memory T cells exit the cell cycle, but do not die. In subsequent infections, memory cells have a shorter time to activation and completion of the first cell division, allowing immune memory to clear infections faster than a naive response. Our analysis suggests that once activated, cells of any generation may return to what the model conveniently refers to as "a resting phase". While we do not know whether cells in the experiment did return to a resting phase after a period of activation, the model suggested that some cells stopped dividing, and those that did so did not appear to die during the experiment because the proportion of dead cells did not increase massively. Molecular mechanisms for CD8+ T cell survival after post-activation cell cycle as predicted by our model fitting have been described. Such mechanisms include expression of the cycling-dependent kinase inhibitor proteins *p*16^*INK*4*a *^in activated, naive CD8 T cells as described by Migliaccio et al [39], and expression of *p*21^*Cip*1 ^and *p*27 as described by Grayson et al [40]. We estimated probability that a cell returns to rest as greater than 0. 20, which is in agreement with these prior experimental descriptions. Our analytic observation may also suggest a mechanism for memory CD8 T cell generation, namely that these "resting" cells may be memory T cells, and that they may arise in each generation after activation. Zand et al [41] have previously hypothesized a similar mechanism for generation of CD4 T cell memory, and Ganusov [42] hypothesized a similar mechanism for generation of memory CD8+ T cells *in vivo *during lymphocytic choriomeningitis virus infection.

A second feature of the proliferation of activated CD8 T cells identified by our model is that the time to death of activated cells calculated from the last cell division appeared to be very short, suggesting that the decision to die is made either shortly after division or even in preceding generations. In the latter case, it would be reasonable to expect that both sister cells could die. Our model does not account for such a possibility, but as discussed previously this class of dependencies does not alter the conclusions based on our model. Such mechanisms of cell differentiation dependency have been described for T lymphocytes, generally involving epigenetic modifications transmitted to daughter cells, but have not been described for transmission of apoptosis from mother to daughter CD8 lymphocytes. Confirming such a novel mechanism of inherited cell death would require further experimental work.

### Open questions

A number of studies have investigated and discussed the merit of a number of mathematical models to describe cell kinetics during CFSE-labeling experiments. Little efforts have been devoted to the development of statistical methods for the analysis of CFSE-labeling data. Their analysis, however, requires specific care as discussed by Hyrien and Zand [12]. The statistical methods proposed by these authors were appropriate only when the prevalence of cell death remains negligible. More work appears needed for when cell death cannot be neglected, which we intend to cover in a separate publication. The branching process model presented herein offers a flexible framework for the analysis of CFSE-labeling experiments. A question that remains open is whether the parameters of models of such complexity are all estimable from CFSE-labeling data. Although our fitting algorithm appeared to converge consistently to the same parameter value, it will be important to develop techniques that help formally determine which parameters can be estimated using CFSE-labeling data.

## Declaration of Competing interests

The authors declare that they have no competing interests.

## Authors' contributions

O.H., R.C., and M.S.Z. developed the model, analyzed the data and wrote the paper; M.S.Z. and O.H. designed the experiment; O.H. and R.C. implemented the approach and studied properties of the model. All authors read and approved the final manuscript

## Reviewers' comments

### Reviewer's report 1

Marek Kimmel, Department of Statistics, Rice University, Houston, Texas, United States

The authors introduce a new variant of the Bellman-Harris Branching Process to improve the mathematical description of the decision-making process in cultured CD8+ T lymphocytes and apply their model to labeling data, to demonstrate its superiority over the competing risks model. The new variant of the process allows the decision to proceed to either to proliferation or to apoptosis (or to quiescence) to precede the act of division or apoptosis initiation. It results in a slightly modified integral equation for the probability generating function of the process. Since the CFSE-labeling allows distinguishing between cells which underwent different numbers of division, as well as quantifying the increase in cell number, fitting models to the CFSE data appears quite meaningful. In my opinion, the paper is worthwhile and publishable. I have several remarks which may improve the contents and clarity of presentation.

1. The process *Z*_*g*,*s*_(*t*) (p. 8) does not seem to be a point process.

**Authors' response. ***We have removed the reference to point processes.*

2. Page 13. The sentence starting from "Clearly, the conditional distribution ..." does not seem to be very precise. I would suggest stating the property discussed in the terms of independence of random variables, so that ambiguity is avoided.

**Authors' response. ***Done.*

3. An application to human CD8+ T lymphocytes. I understand that in this application, independence between mother and daughter lifetimes is assumed. This might be worth stressing, particularly since one page earlier, in Proposition 1, independence is not required.

**Authors' response. ***Done.*

4. Data analysis using a competing branching process. I find the section written in a somewhat confusing way. Among other, it is first stated that "A likely explanation for the lack of fit of the competing risk process is that the times to death were estimated as being much shorter than the times to division using the full model." However, several lines down, the mean time to division is said to be estimated as 14.3 hours whereas the mean time to death as 14.7 hours. Please clarify.

**Authors' response. ***We have re-written the section.*

5. Effects of cellular dependencies. There exists a whole class of models, developed mainly in the 1980s, which look for the source of dependencies in cell size/growth rate regulation. A relatively complete review is contained in Webb's paper in Computers & Mathematics with Applications (Volume 18, Issues 10-11, 1989, Pages 973-984).

**Authors' response. ***The reference has been added.*

6. Further quantitative insights into the kinetics of CD8 T cells. It seems somewhat disappointing that this discussion does not return to the original motivation, which was the organization of fighting the infection by the T lymphocytes. Maybe something might be added?

**Authors' response. ***We have expanded the corresponding paragraph.*

### Reviewer's report 2

Wai-Yuan Tan, Department of Mathematical Sciences, The University of Memphis, Tennessee, United States

This paper proposed an age-dependent branching process model for analyzing data from CFSE-labeling experiments, extending the multi-type Bellman-Harris branching process and the author's previous models. The paper is well-written and provides a logical approach to model the temporal evolution of population of cells under cell division, differentiation and death. The paper also went into details to illustrate how to implement the proposed model. It has also identified some basic cellular dependencies that may influence the expectations of the process. Because the model involves a large number of unknown parameters, it would be very useful to the readers if the authors would write a paragraph to indicate how the unknown parameters and probabilities were estimated. What type of methods they used to derive standard errors of the estimates (Efron's bootstrap method?). Because some of the distributions are basically mixtures, perhaps the EM-algorithm may be useful.

### Authors' response

Parameter estimation using CFSE-labeling data is a difficult problem that will be discussed in a separate paper. In particular, it can be shown that the proposed estimator is root-n consistent, whereas some existing estimators are not. Also, Efron's bootstrap does not appear to apply to CFSE-labeling experiments because the resulting data are dependent. Moreover, to the best of our knowledge, existing bootstrap algorithms do not appear to have been designed to handle this class of dependencies. Finally, as you suggested, one can used an EM algorithm to compute parameter estimates.

### Reviewer's report 3

Peter Olofsson, Mathematics Department, Trinity University, San Antonio, Texas, United States

The paper uses a multi-type age-dependent branching process to model CFSE-labeling experiments. It is a clear and well-written piece of work and I think it deserves to be published in Biology Direct. I do not have the necessary expertise to comment on the biology, and will only make a few comments on the mathematics. On page 7, the authors claim to extend the Bellman-Harris process by allowing lifespan and offspring to be dependent. However, such processes were introduced already in the 1960s by B.A. Sevastyanov and are sometimes referred to as "Sevastyanov processes". These days, a Sevastyanov process can be viewed as a special case of the general multi-type branching process introduced by Peter Jagers in the late 1980s and early 1990s. I would therefore recommend the authors not to refer to their process as "the general branching process" as this term is commonly reserved for the "Crump-Mode-Jagers process" where reproduction occurs according to a point process, not necessarily by splitting.

### Authors' response

We agree that branching processes allowing dependencies between cell fate and cell lifespan are not new, as noticed in some of our past publications [14,19]. The use of the terminology "general branching process" is now avoided.
